# Re: Choices have consequences: the nexus between DNA repair pathways and genomic instability in cancer 

**DOI:** 10.1186/s40169-017-0137-6

**Published:** 2017-02-07

**Authors:** Michael McKay

**Affiliations:** University of Sydney, Sydney, Australia

To the Editor,

Despite a broad DNA repair title implicating defective repair with genomic stability and cancer, this paper focuses largely on repair of DNA double strand breaks (DNA DSBs) by one of the two main mechanisms for their resolution—homologous recombination (HR). It is important for those not in the field to be aware of the many other DNA lesion types associated with cancer, including pyrimidine dimers induced by ultraviolet radiation and oxidative nucleotide damage induced as biproducts of cellular metabolism as well as exogenous DNA damaging agents. Likewise, canonical DNA repair pathways and subpathways, apart from HR, are associated with cancer predisposition. These include defective nucleotide excision repair in xeroderma pigmentosum (massive increase in skin cancer risk) [1] and mismatch repair in hereditary non-polyposis colon cancer [2]. These associations have been extensively reviewed [3]. Base excision repair may also have a role in cancer avoidance via epigenetic protection of CpG [4] islands and is a major therapeutic target in specific cancer types [5].

The Future Perspectives summary paragraph also states that DSBs are caused by chemotherapy. While this is true for the minority of chemotherapeutic agents, it is characteristic of ionizing radiation, used as radiotherapy in around one half of all cancer patients in the Western World [6]. It is important to note that modulation of DNA DSB repair in cancer may have very significant implications for this form of cancer therapy.
